# Data synthesis for crop variety evaluation. A review

**DOI:** 10.1007/s13593-020-00630-7

**Published:** 2020-07-09

**Authors:** David Brown, Inge Van den Bergh, Sytze de Bruin, Lewis Machida, Jacob van Etten

**Affiliations:** 1 Laboratory of Geo-Information Science and Remote Sensing, Wageningen University & Research, Droevendaalsesteeg 3, 6708 PB Wageningen, The Netherlands; 2 Bioversity International, Turrialba, 30501 Costa Rica; 3 Bioversity International, C/O KU Leuven, W. De Croylaan 42, P.O. Box 2455, 3001 Leuven, Belgium; 4 Bioversity International, C/O International Institute of Tropical Agriculture (IITA), Nelson Mandela African Institute of Science and Technology, P.O. Box 447, Arusha, Tanzania

**Keywords:** Data-driven decision-making, Meta-analysis, Multi-environment trials, On-farm trials, Data repurposing, Environmental data, Farmers’ preferences

## Abstract

Crop varieties should fulfill multiple requirements, including agronomic performance and product quality. Variety evaluations depend on data generated from field trials and sensory analyses, performed with different levels of participation from farmers and consumers. Such multi-faceted variety evaluation is expensive and time-consuming; hence, any use of these data should be optimized. Data synthesis can help to take advantage of existing and new data, combining data from different sources and combining it with expert knowledge to produce new information and understanding that supports decision-making. Data synthesis for crop variety evaluation can partly build on extant experiences and methods, but it also requires methodological innovation. We review the elements required to achieve data synthesis for crop variety evaluation, including (1) data types required for crop variety evaluation, (2) main challenges in data management and integration, (3) main global initiatives aiming to solve those challenges, (4) current statistical approaches to combine data for crop variety evaluation and (5) existing data synthesis methods used in evaluation of varieties to combine different datasets from multiple data sources. We conclude that currently available methods have the potential to overcome existing barriers to data synthesis and could set in motion a virtuous cycle that will encourage researchers to share data and collaborate on data-driven research.

## Contents

- 1. Introduction
- 2. Data required for crop variety evaluation
  - 2.1 Agronomic performance data
  - 2.2 Environmental data
  - 2.3 Food quality and consumer preference data
- 3. Data management challenges
  - 3.1 Main barriers for data availability and integration
  - 3.2 Efforts to overcome data management problems
  - 3.3 Further work to improve data availability and data integration
- 4. Analysis of different types of data
  - 4.1 Multi-environment trial analysis
  - 4.2 Evaluation in target environments and including user requirements
  - 4.3 Multi-dimensional assessment for decision-making
- 5. Data synthesis approaches
  - 5.1 Rank aggregation methods
  - 5.2 Network meta-analysis
  - 5.3 Assessment of available data synthesis methods
    - 5.3.1 Partial overlapping among evaluated accessions across trials
    - 5.3.2 Measurements based on different rating scales or different methods
    - 5.3.3 Relative strengths and weaknesses of data synthesis methods
- 6. Conclusions and recommendations
  - 6.1 Elements for a data synthesis approach are available and aligned around ranks and reliability
  - 6.2 Data synthesis should progress from general to specific and from simple to complex
  - 6.3 Use cases can spur further data sharing and model development
  Acknowledgements
  References

## Introduction

Farmers, especially smallholders in developing countries, are facing ever more challenging production conditions and product requirements. Extreme weather events are on the rise as one of the effects of climate change (Lesk et al. 2016; Coumou and Rahmstorf 2012). Emerging pests and diseases, as well as declining soil fertility, are also constraining farm productivity. Evolving crop production practices require the development of new genotypes that meet specific agronomic traits (Collard and Mackill 2008). Markets are also evolving, and taste preferences need to be considered if new crop varieties are to easily find their way to the consumer (Dawson and Healy 2018). Furthermore, there is growing knowledge of the different product needs and preferences relative to gender, which are influenced by their different roles in the value chain, differences in access to land and other inputs and differences in decision-making power (Christinck et al. 2017). There is also an increasing interest in more sustainable crop production systems, which would require a redesign of the whole food system and the role of players involved, including breeders (Lammerts van Bueren et al. 2018). Crop improvement aims to address the multiple challenges faced by farmers through delivering improved varieties (Malosetti et al. 2013). However, simply using the most recently released variety will not always lead to improvement, as breeding cannot address all requirements in all contexts. Decision-makers involved in crop improvement, including breeders, agronomists and farmers, evaluate multiple aspects and trade-offs relevant to the context in which they use the varieties. Crop variety evaluation is critical in decision-making in crop variety release, crop seed marketing or distribution and generating crop variety recommendations for farmers.

**Fig. 1:**
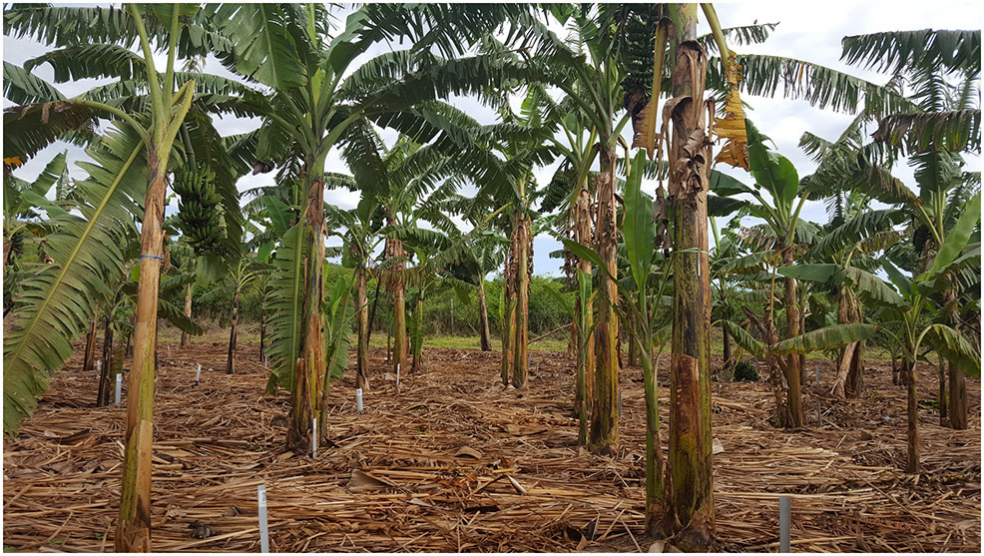
NARITA hybrid field trials in Mbarara, Uganda, mulched with swamp grass to reduce weeds and soil moisture loss. Photo credit: Bioversity International/L. Machida

Crop variety evaluation is mainly conducted through field trials (Fig. 1), which are expensive and time-consuming (Lecomte et al. 2010; Tenkouano et al. 2012; Kipp et al. 2014). The limitations in resources, space, time and the required logistics in field trials also make it almost impossible to test all the varieties of interest in the same trial or in all the possible environments (Simko et al. 2012; Singh et al. 2014; Lecomte et al. 2010). Crop variety evaluation usually considers yield as the main trait while disease resistance and climate adaptation are secondary traits. Other characteristics of interest in crop variety evaluation, such as product quality and consumer preferences, are obtained through quality assessments and sensory evaluations, which are also expensive (Tomlins et al. 2004). An exception in terms of costs of data relevant to crop variety evaluation is climatic data, which acquiring costs have been decreasing due to advances in remote sensing and computational power.

Crop variety evaluation has not always kept pace with the growing complexity of agricultural production and the growing availability of data. As a data-driven type of research, crop variety evaluation can benefit from multiple revolutions occurring in several fields such as genomics, phenomics, big data and machine learning (Bolger et al. 2019; Esposito et al. 2020; van Etten et al. 2017; Tardieu et al. 2017). These revolutions are driven by increased data storage and computing capacity, the availability of sensors, improved DNA sequencing technologies and new field data collection approaches, such as high-throughput and high-precision field phenotyping and crowdsourcing (Tardieu et al. 2017; Esposito et al. 2020; Chawade et al. 2019; Reynolds et al. 2020; Van Etten et al. 2016). This has caused not only a quantitative leap in data volumes but also a shift to ‘big data’ approaches that move beyond small-sample statistics to data analysis based on machine learning (Breiman 2001; Thessen 2016; van Etten et al. 2017; Ersoz et al. 2020). While there are multiple examples of useful applications of big data analysis in agriculture (Kamilaris et al. 2017; Liakos et al. 2018), such cases are still few compared to other industries (Kamilaris et al. 2017).

Specifically, crop variety evaluation has taken little advantage of the potential benefits of data synthesis. Data synthesis allows the combination of data from different sources, producing new information and knowledge to support decision-making (Pillemer and Light 1980; Pickett et al. 2007; Carpenter et al. 2009; Wyborn et al. 2018).

**Fig. 2:**
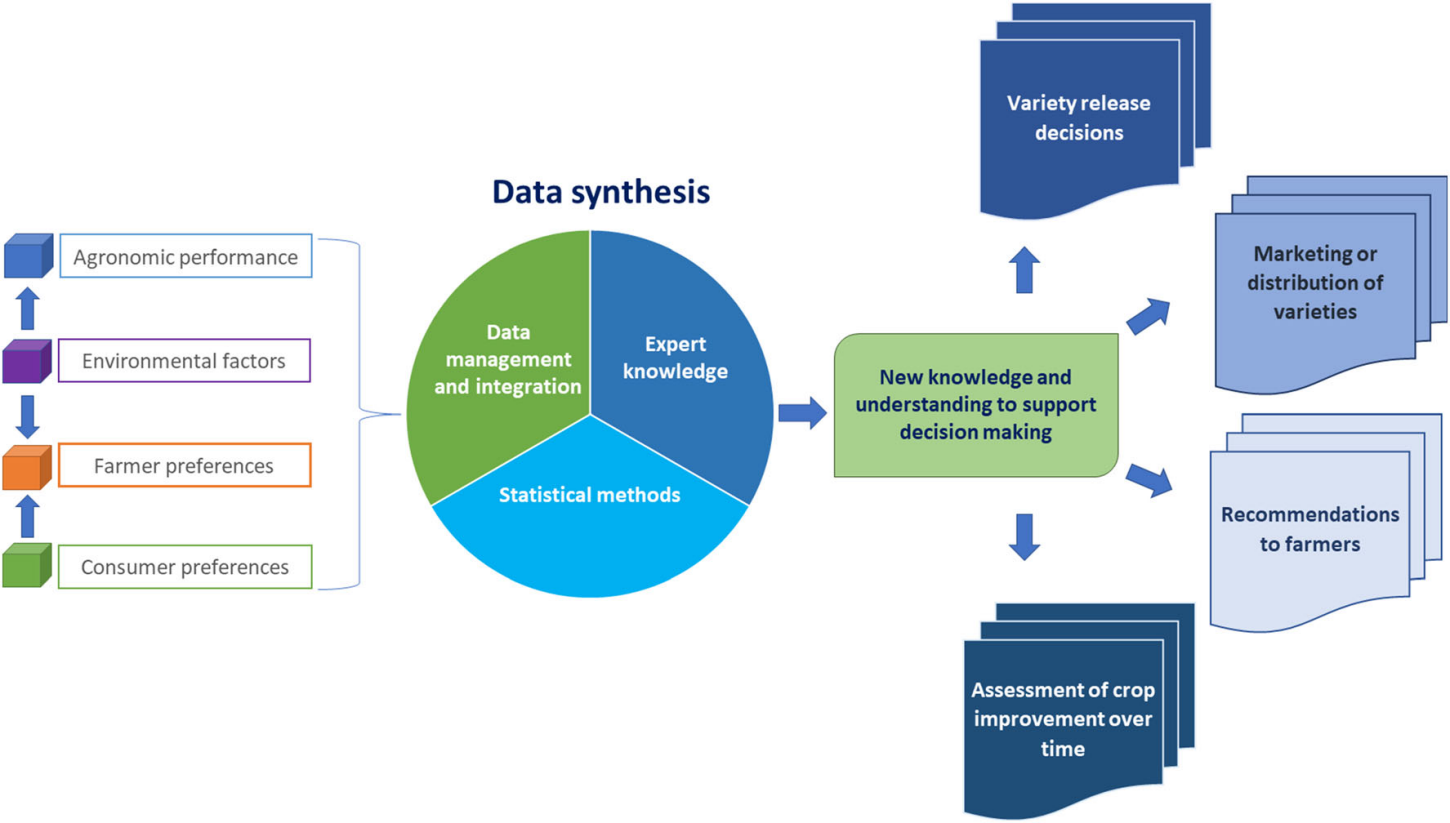
Different elements and processes involved in data synthesis for crop variety evaluation

Interest in the value from combining and (re)using datasets in agriculture has grown, supported by open data and data sharing initiatives (Leonelli et al. 2017). As new analytical technologies and methods become available, legacy data could be reanalysed (White and van Evert 2008; Hampton et al. 2013). Data synthesis can improve the efficiency of data use in crop variety evaluation by combining and repurposing new and legacy data from field trials, environmental measurements, farmer requirements and consumer preferences (Fig. 2). In the agricultural sciences, it has so far mainly taken the form of meta-analysis (Philibert et al. 2012; Krupnik et al. 2019).

Data synthesis can play a role in different functions of crop variety evaluation. The selection of genotypes to be released as cultivars can benefit from data synthesis to assess genetic gain (progress over time) (Streck et al. 2018), to benchmark against other breeding programs, to improve accuracy through multi-season assessments and to predict performance beyond the trial environments. The latter involves analysing a combination of variety trial data and environmental data (Hyman et al. 2013). The analysis of trial data can be made more accurate when data from the last trial season is combined with historical variety performance data (Arief et al. 2015).

To release a new variety, breeders need to evaluate the proposed genotypes against existing varieties in a country or region. Data synthesis could facilitate enriching data from trials including the new varieties with data on the past performance of the older varieties to gain accuracy (Damesa et al. 2017). Seed companies need to assess variety performance to take seed production and marketing decisions. Service providers, such as agro-input suppliers, cooperatives, agricultural extension organizations and NGOs, need to make recommendations to farmers, considering the multiple dimensions of variety performance (and trade-offs between these dimensions) in different environments and under different types of crop management. Information from existing crop trials to formulate recommendations is often used for this end. Such an analysis of existing data could benefit from data synthesis if the data that is available comes from different sources. Some service providers produce their own data about on-farm variety performance to generate recommendations or refine existing ones, which could also benefit from data synthesis to combine the new data with existing data. In many contexts, variety evaluation is done in a fragmented way (Rangarajan 2002), which can preclude centralized coordination or standardization of data collection and weakens variety evaluation as each entity assesses genotype by environment interactions in a limited set of environments. Data synthesis could help to gain a better understanding of genotype by environment interactions across space and time. Flexible data synthesis could take advantage of heterogeneous data from different actors in the seed sector and provide value to the several functions that variety evaluation plays in each step of the crop improvement cycle.

A clearer perspective on data synthesis for crop variety evaluation is needed to achieve these potentials. Here, we review the literature relevant to data synthesis for context-specific decision-making in variety management. The objective of this article is to provide an overview of the required elements, current approaches and research gaps in data synthesis for crop variety evaluation, focusing on decision-making for variety pre-release and post-release. We limit ourselves to these later stages of the breeding process, and therefore, we do not cover the genomic and high-throughput phenotyping data. Even though these types of data are clearly part of the data revolution in crop improvement, they generally concern early and intermediate stages of the breeding process. We briefly refer to high-throughput field phenotyping data, as it has the potential to support later stages of the breeding process. In Section 2, we discuss the types and sources of data that are required. In Section 3, we discuss how data synthesis relies on proper data management, including sharing data across different trials and the compatibility of datasets. Data synthesis requires not only combining datasets to assess variety performance but also beyond assessing average performance, a careful analysis of how different genotypes respond to diverse environments and match the preferences of farmers, consumers and other stakeholders. Therefore, in Section 4, we review how data analysis is currently dealing with the end-users, their context and what is still lacking to evaluate crop varieties through a data synthesis approach. In Section 5, we review existing data synthesis approaches used in crop improvement and assess how they can be enhanced to include use context. In Section 6, we present our conclusions and recommendations.

## Data required for crop variety evaluation

In this section, we describe the data types required by a data synthesis approach for crop variety evaluation. Field trial data are important to analyse the phenotypic response of a given genotype, to the environmental characteristics of the testing location and, in some cases, to management practices. Not only yield but also product quality is considered in variety evaluation. The evaluation of crop varieties also involves data about the preferences of farmers obtained from participatory and on-farm trials, and consumers, obtained from sensory evaluations.

### Agronomic performance data

Agronomic performance data are collected from field trials, which can be set up in several ways depending on the context and purpose. A rough classification of contexts includes (1) public international breeding programs (e.g. breeding programs within the CGIAR, (2) private breeding programs at commercial seed companies and (3) agricultural research at national or regional level, conducted by National Agricultural Research Systems, often in partnership with International Agricultural Research Organizations.

Field trials of breeding programs are usually known as performance trials or yield trials, given the importance of yield as the main trait (Acquaah 2012). There are two main types of yield trials: (1) breeder trials and (2) official trials (Acquaah 2012). Breeder trials aim to assess the performance of a set of genotypes to decide which ones should be released as cultivars (Priyadarshan 2019). An official variety trial is part of the variety release and registration process, which varies among countries, but in most of the cases, it is conducted by an independent body, such as an official seed agency or under the jurisdiction of a variety release committee. Depending on the stage of the breeding process, the breeder’s trials can be divided into preliminary yield trials (PYTs) and advanced yield trials (AYTs) (Priyadarshan 2019). A PYT often concerns many genotypes (and few replications), whereas an AYT evaluates a small number of genotypes (selected from the PYT), with more replications over different environments, and during several years (Priyadarshan 2019). In this review, we are focusing on data generated from AYT. Crop variety trials can also be established to test improved varieties to be recommended to farmers (Yan 2014b). Both for breeding and to generate variety recommendations, field trials aim to evaluate varieties in different environments, where the environment is considered a combination of location and season (Acquaah 2012). For this purpose, crop variety trials can be established mainly in three different levels of combination of location and season: (1) a single location in a single season, (2) multiple locations in a single season and (3) multiple locations in multiple seasons (Yan 2014a). Crop variety trials can also be established in a centralized or decentralized system. The centralized approach involves on-station trials; it is the conventional approach for many crops and contexts. Decentralized methods include establishment of trials at farm locations, with different levels of participation from farmers. As not only biophysical factors determine the suitability of a variety, it has been suggested that the concept of environment should be extended to include the socioeconomic context of the target location (Desclaux et al. 2008). Participatory plant breeding and participatory varietal selection methods aim to better consider farmers’ preferences and context in order to increase the adoption of improved varieties (Weltzien and Christinck 2017; Ceccarelli and Grando 2007). These approaches often include participatory on-farm trials, which may produce insights that are complementary to insights derived from conventional trials (Coe 2007; Coe 2002; Atlin et al. 2001). Although farmers’ participation is commonly associated with on-farm trials, farmers can also be involved in on-station trials. Participatory varietal selection (PVS) can be done through mother-baby trials, where the mother trial includes the full set of testing genotypes and the baby trial only includes a subset of test genotypes alongside the control genotype (Virk et al. 2009; Snapp 2002). On-farm trials may produce unbalanced data, due to differences in the particular conditions of farmers’ fields and the limited availability of seeds of the new varieties (Virk et al. 2009).

Data collected from field trial evaluations typically include trial design, the trial location, the date of establishment, trial management, evaluated genotypes and observations of the target traits (e.g. yield). Observations of the target trait can be either measured or estimated and should be ideally referenced to the observation date and to the phenological stage of the plant during observation (Billiau et al. 2012; White et al. 2013; Germeier and Unger 2019). For instance, the second phase of the International Musa Testing Program (IMTP) used the following attributes (Orjeda 2000): genotype, time from planting to shooting (days), time from shooting to harvest (days), time from planting to harvest (days), height of the pseudostem at shooting (cm), height of the following sucker at harvest (cm), bunch weight (cm), number of hands per bunch (hands), total number of fingers per bunch (fingers), average fruit weight (g) and leaf emission rate.

Technological innovations allowed the development of new methodologies for collecting data from field trials. These include high-throughput field phenotyping methods supported by satellite imagery or data from unmanned aerial vehicles (UAVs) and proximal phenotyping (Chawade et al. 2019).

Given the multiple context and evaluation objectives, each organization conducting crop variety trials may use its own experimental design and employ different methods and technologies for collecting, storing and publishing and/or sharing data. In Section 3, we review potential obstacles for data integration resulting from these differences. Furthermore, the diversity of goals and evaluation methodologies also produce different approaches to analyse collected data. We review these in Section 4.

### Environmental data

To analyse the phenotypic response of genotypes to the testing environment, a fundamental step is to characterize the environment. The environment is the first source of yield variability in plant breeding trials (Chenu 2015). Hence, environmental data are required to characterize the trial location and to understand its influence on the performance of tested genotypes in that particular location. It is known that the use of environmental data as model covariates analysing multi-location trial data improves the degree of accuracy in the prediction of genotype performance (Piepho 2000; Piepho et al. 1998). A recent study by van Etten et al. (2019) demonstrated an improvement in variety recommendations using seasonal climate data as model covariates. Xu (2016) proposed to consider all environmental factors affecting growth and production of plants through an approach called ‘envirotyping’. Environmental data can be collected at trial sites directly. But even if environmental data were not collected during trials, geolocating trial sites allows enriching the dataset with existing environmental data. Adding environmental data to legacy trial data allows comparisons among trials conducted at different locations. Some types of environmental data are increasingly available through open and public repositories (Hyman et al. 2013). For instance, data on rainfall, temperature, elevation and soils are openly and freely available from open and public databases such as Climate Hazards Group InfraRed Precipitation with Station data (CHIRPS) (Funk et al. 2015), MODIS Land Surface Temperature (Wan et al. 2015), Hole-filled SRTM for the globe version 4 (Jarvis et al. 2008) and SoilGrids (Hengl et al. 2017). The European Centre for Medium-Range Weather Forecasts (ECMWF), through the Copernicus Climate Change Service (C3S), provides a comprehensive collection of climatic datasets, including the recently deployed ‘Agrometeorological indicators from 1979 to 2018 derived from reanalysis’, known as AgERA5. Available climatic data can be used to calculate climatic indices (Table 1), which were proven to be useful as model covariates in the analysis of crop variety trials (van Etten et al. 2019; Kehel et al. 2016). Even though climatic data opens a wide range of possibilities for crop variety evaluation, the resolution of available data has to be carefully considered (Parkes et al. 2019).

**Table 1:** Temperature and precipitation indices commonly used as covariates in crop variety trial analyses

Environmental index	Unit
Maximum daytime temperature	°C
Minimum daytime temperature	°C
Maximum nighttime temperature	°C
Minimum nighttime temperature	°C
Mean difference between daily maximum temperature and daily minimum temperature	°C
Number of days with maximum temperature > 30 °C	Days
Number of nights with maximum temperature > 25 °C	Days
Maximum length of consecutive days with precipitation < 1 mm	Days
Maximum length of consecutive days with precipitation ≥ 1 mm	Days
Number of days with precipitation > 5 mm	Days
Number of days with precipitation > 10 mm	Days
Maximum 1-day precipitation	mm
Maximum 5-day precipitation	mm

Adapted from van Etten et al. (2019) and Kehel et al. (2016)

### Food quality and consumer preference data

Sensory and nutritional evaluation has received more attention in recent decades, countering the narrow focus of crop improvement on yield, disease resistance and uniformity (Folta and Klee 2016). At present, consumer markets are evolving, with consumers seeking additional product qualities such as nutritional and sensorial characteristics (Folta and Klee 2016). Food quality involves both objective and subjective analyses, involving measurements of contents, texture as well as sensory analyses. Sensory evaluation is formally defined as ‘a scientific discipline used to evoke, measure, analyze, and interpret reactions to those characteristics of foods and materials as they are perceived by the senses of sight, smell, taste, touch, and hearing’ (Anonymous 1975; Stone et al. 2012). Consumer preference data are obtained from sensory evaluations by panels of regular or specialized consumers, with different methods, such as descriptive analysis or rapid sensory evaluations (Dawson and Healy 2018). Sensory and hedonic (i.e. related to pleasant or unpleasant) experiences cannot be measured directly and should be inferred from descriptive or numerical representations (hedonic scales) of subjects’ responses (Lim 2011). There are four main types of scales used in hedonic scaling, which are presented in Table 2.

**Table 2:** Types of hedonic scales

Scale	Basic empirical operation	Number usage	Permissible statistics	Example scale
Nominal	Determination of equality (categorization)	As labels	Non-parametric: number of cases; mode	1 (good)
				2 (bad)
Ordinal	Determination of greater or less	To recognize the rank order	Non-parametric: median; percentiles	Rank rating
Interval	Determination of equality of intervals or differences	To represent degrees of differences	Parametric: mean; standard deviation	Equal intervals
Ratio	Determination of equality of ratios	To represent relative proportions	Parametric: log mean; standard deviation	Labelled affective magnitude (LAM) scale; labelled hedonic scale (LHS)

Adapted from Stevens (1946) and Lim (2011)

The 9-point hedonic scale developed by Peryam and Girardot (1952) is the most widely used method for scaling consumer preference and food acceptability (Lim 2011). It is composed of the following values and their correspondent description: 9, like extremely; 8, like very much; 7, like moderately; 6, like slightly; 5, neither like nor dislike; 4, dislike slightly; 3, dislike moderately; 2, dislike very much; and 1, dislike extremely (Peryam and Girardot 1952). More recently, other scaling methods have been proposed, such as the labelled affective magnitude (LAM) scale (Schutz and Cardello 2001) and labelled hedonic scale (LHS) (Lim et al. 2009). This diversity of measurement scales can pose a challenge to combine data from different sources, such as different laboratories testing food quality, or a sensory evaluation with farmers testing different varieties as part of a breeding process.

Agronomic performance, food quality and preference data are still expensive and complex to acquire and manage. In contrast, weather and soil data are increasingly available at significantly reduced costs. More effort is required to improve the efficiency in data use in the evaluation of crop varieties. The higher availability of weather and soil data can motivate and increase data reuse, repurposing legacy crop variety trial data by adding environmental data to extract new insights.

## Data management challenges

As a data-driven research, data synthesis requires availability of the data to be reused. It also requires careful data management to integrate data of heterogeneous nature from different sources and formats, as described in Section 2. Here, we discuss the challenges in data management that are relevant to data synthesis for crop variety evaluation, the main efforts to address these problems and further research needs.

### Main barriers for data availability and integration

Data should be available to be integrated and then synthetized using formal statistical techniques. However, data are still rarely shared for reuse, especially in the case of raw data from variety trials. Diekmann (2012) and Williams (2012) found that researchers are often unwilling to share raw data out of concern about data being taken out of context, which could lead to incorrect results and misinterpretation. Authors may also oppose freely releasing data that cost them substantial work and resources, whereas they may be more willing to share data with colleagues (Diekmann 2012).

In addition to cultural constraints, technical challenges arise for data sharing among both individuals and institutions. It has been a common practice for researchers and research centres to develop their own system for storing data, mainly because they do not trust global repositories with which they do not have a direct relationship and for which long-term support may not be guaranteed (Leonelli et al. 2017). This has resulted in a myriad of individual databases that are neither open nor compatible among research centres. This not only inhibits collaboration among scientists but also promotes duplication of efforts and increases costs, a luxury that the scientific community cannot afford in times of scarce economic resources for agricultural research.

In cases when data are available, data integration sometimes encounters problems due to lack of standardization in terms of syntax, semantics and structure. Crop variety trial datasets are often very heterogeneous in terms of quantity, quality, types and formats (Hyman et al. 2017; Leonelli et al. 2017). Individual trial designs and observational methods vary according to the specific purpose of trials (Section 2). This lack of standardization of crop variety trial data makes it difficult to compare results between trials and to reuse datasets with traditional tools (Rijgersberg and Top 2000; Leonelli et al. 2017). Combining crop trial datasets often presents the following problems: (1) incomplete or inexistent overlap among evaluated accessions across trials, (2) measurements based on different rating scales and (3) the use of different methods for observing the same trait (Simko and Pechenick 2010). Methods to solve the problem of measurement in different rating exist such as the threshold model (Hartung and Piepho 2005), but it does not solve the problem of partial overlap among tested varieties in the different trials. In Section 5, we explore and evaluate how different data synthesis methods deal with this kind of problems.

The dearth of relevant data in the public domain limits the possibilities of data synthesis, as it provides a large initial cost of assembling, cleaning and reformatting data. For individual data synthesis efforts, this initial investment may be relatively very high, even though it could be worthwhile if data can be repurposed more than once. Furthermore, practices limiting data reuse go against the aim of science of building universal knowledge, in which public funds play a fundamental role.

### Efforts to overcome data management problems

Several international agricultural research organizations have made efforts to address the barriers of poor data availability and data format incompatibilities (McLaren et al. 2005; Ritchie 1995; Germeier and Unger 2019). Several global funders of agricultural research are increasingly seeking mechanisms to guarantee that research investments generate benefits for smallholder farmers in developing countries (Dalrymple 2008). Global open data and sharing initiatives aim to facilitate data accessibility, allowing the generation of new knowledge (Wilkinson et al. 2016). With valuable contributions from diverse partner organizations, CGIAR centres have been developing information systems and platforms, aiming to integrate heterogenous data sources to support crop improvement research (McLaren et al. 2005; Hyman et al. 2017). Examples of this type of systems are the International Crop Information System (ICIS) and its derivatives, the International Rice Information System (IRIS) and the International Maize Information System (McLaren et al. 2005; Shrestha et al. 2010). The recently created CGIAR ‘Platform for Big Data in Agriculture’ aims to materialize the potential of big data–related methods and technologies to improve agricultural production. Outside the CGIAR system, agricultural researchers and organizations are also endeavouring to construct better ecosystems of data and methods. Table 3 contains a compilation of the main international initiatives on standardization and data sharing. Data standardization and sharing systems include online databases such as AgTrials, YamBase, CassavaBase and MusaBase, which all implement ontologies to standardize vocabularies and terminologies (see Table 3). Ontologies formally define the relationships among concepts within a given domain (Matteis et al. 2013). Similar approaches have also been proposed by other authors. For instance, Rijgersberg and Top (2000) proposed data model templates—a generalization of data models—to achieve a balance between standardization and flexibility. See Spyns et al. (2002) for an overview of specific differences between data models and ontologies. Germeier and Unger (2019) applied a modelling approach that goes further than data models, considering also statistical models in the implementation of a phenotyping information system. Efforts to standardize phenotyping data include the Minimal Information About Plant Phenotyping Experiment (MIAPPE) (Krajewski et al. 2015; Ćwiek-Kupczyńska et al. 2016; Papoutsoglou et al. 2020). Efforts to standardize data from field experiments include the International Consortium for Agricultural Systems Applications (ICASA) standard, initially developed by the International Benchmark Sites Network for Agrotechnology Transfer (IBSNAT) and updated by ICASA (White et al. 2013).

**Table 3:** Main international initiatives to increase data sharing and standardization in agriculture

Initiative	URL
AgTrials	http://agtrials.org
AgroPortal	http://agroportal.lirmm.fr
Breeding Application Programming Interface (BrAPI)	https://brapi.org
Breeding Management System	https://bmspro.io
CassavaBase	https://www.cassavabase.org
Crop Ontology	http://www.cropontology.org
GARDIAN	http://gardian.bigdata.cgiar.org
Global Open Data for Agriculture and Nutrition (GODAN)	https://www.godan.info
Global Trial Data Management System	https://research.cip.cgiar.org/gtdms
Integrated Breeding Platform	https://www.integratedbreeding.net
MIAPPE	https://www.miappe.org
MusaBase	https://musabase.org
Sol Genomics Network	https://solgenomics.net
YamBase	https://yambase.org

The FAIR (findability, accessibility, interoperability and reusability) guiding principles are intended for producing and publishing data, aiming to facilitate and enable data sharing and reuse (Wilkinson et al. 2016). To achieve these principles, a set of mechanisms is considered. These include unique and persistent identifiers, a standardized communications protocol and the use of domain-specific standards for both data and meta-data.

Despite these efforts, recent literature indicates that there are still serious challenges in implementing data standardization and sharing. About 85% of the more than 35,000 records in the AgTrials database contain only meta-data; hence, those interested in the underlying data should contact the original data provider (Hyman et al. 2017). Problems persist on both the supply and demand side. It has been found that researchers are often reluctant to use data produced by others because there is no guarantee about the quality (Diekmann 2012). Data standards are now available (Table 3), but it is still difficult to persuade the agricultural research community to adopt the suggested standards. The lack of flexibility to adapt to scientific progress is indeed one of the arguments stated against standards (Germeier and Unger 2019). For both new and legacy data, standardization requires considerable efforts, which will not immediately pay off, hindering its implementation. In addition, the efforts required for processing datasets with the accompanying meta-data to facilitate open access and use by others are often not acknowledged (Diekmann 2012; White and van Evert 2008). From 112 surveyed users of the AgTrials platform, 34% considered that their data are incorrectly organized to be shared publicly (Hyman et al. 2017). In the cases where data are shared, it is mostly not through global repositories but rather as supplementary data on the journal website where associated articles were published (Williams 2012). Sharing data as supplementary materials on a journal website would be adequate if journals followed commonly agreed guidelines, such as the FAIR principles. Although there has been an increase in data shared by researchers during the recent years, there is a still a lack of awareness and hence compliance with FAIR principles (Mark et al. 2018).

### Further work to improve data availability and data integration

**Fig. 3:**
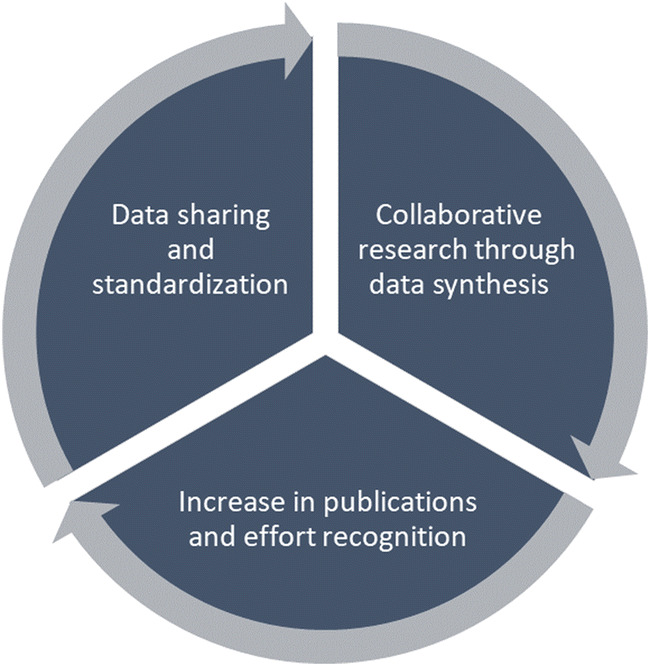
A virtuous cycle set in motion by data synthesis

We identified barriers for data availability and data integration. The main efforts to stimulate data open access and repurposing have focused on compliance with standards and data sharing as a goal. Given the modest progress so far, we suggest that this focus be complemented with efforts to make data sharing more appealing, by the stimulation of data demand for data synthesis. This might set in motion a virtuous cycle of collaboration around data synthesis, providing clear incentives in the form of authorships on joint publications and citations to datasets (Fig. 3).

Therefore, we posit a need for simple methods that can deal with highly heterogeneous datasets to start to show the potential benefits of data synthesis (see Section 5). Ecology research is a concrete example of how increasing the number of datasets, publications and collaboration among larger groups of scientists through meta-analysis can result in even larger collaborative initiatives which enhance the scope and potential impact of research (Cadotte et al. 2012). Data journals may help to boost this kind of data-centred collaboration (Candela et al. 2015). This could provide further motivation and feedback to continue the consensus building processes around data management.

## Analysis of different types of data

Data synthesis requires the combination and analysis of different types of data as described in Section 2. Here, we review current approaches to combine those types of data and research gaps to achieve data synthesis for crop variety evaluation.

### Multi-environment trial analysis

As we discussed in Section 2, the most basic evaluation of genotypes is the assessment of their agronomic performance, such as plant growth parameters, yield, plant reaction to diseases and tolerance to climate conditions (e.g. tolerance to drought, cold and flooding), among others. This type of evaluation is conducted through field trials, which can be established in different ways. Conventional trials are usually established in research stations under controlled conditions. Trials can also be established on farms and involve different levels of farmer participation, from limited participation as observers to full participation as citizen scientists (Ceccarelli et al. 2009; Ceccarelli 2012; Van Etten et al. 2016). Combinations are also possible, such as on-station trials with some level of farmer participation. Regardless of the trial design, the idea is to establish trials at different locations for several growing seasons. The combination of location and time is known as the ‘testing environment’, and the trials are known as ‘multi-environment trials’ (METs). Multi-environment trials are conducted to evaluate the suitability of crop genotypes for different agroecological conditions (van Eeuwijk et al. 2005).

Genotype × environment (G × E) interaction is the relative difference in phenotypic response that a group of genotypes expresses depending on the environmental conditions (de Leon et al. 2016). Hence, G × E assessment requires the evaluation of a minimum of two different genotypes in at least two different environments (Kang 1997). The phenotypic response of a genotype to the environment is described by a function known as the reaction norm (Bustos-Korts et al. 2019). When the reaction norm lines of evaluated genotypes in different environments are not parallel, there is presence of G × E (Bustos-Korts et al. 2019). Especially in conventional breeding, G × E is considered a challenge by breeders due to its implications for genotype selection (Kang and Gorman 1989). Aiming for more specific adaptation, decentralized breeding programs take advantage of G × E instead of diminishing the effects (Ceccarelli 1989).

There are different statistical models for G × E analysis. For an overview, we refer to recent reviews such as the work of Malosetti et al. (2013), van Eeuwijk et al. (2016) and Bustos-Korts et al. (2019). Most of the statistical models for G × E analysis can be interpreted as (phenotypic) response functions for each genotype to environmental variables (van Eeuwijk et al. 2005). Therefore, G × E analysis represents a combination of two of the data types presented in Section 2: the agronomic performance data and the environmental data.

Data from multi-environment trials are usually summarized in two-way tables of means, with genotypes as rows, and environments as columns (Malosetti et al. 2013). Two major groups of statistical models for the analysis of G × E can be identified: (1) methods that only use the two-way table of means and environmental information are included only implicitly (usually as dummy variables) and (2) methods that use additional information, explicitly included as genotype and/or environment covariates (temperature, rainfall, etc.) (Malosetti et al. 2013). Examples of G × E models from the first group include additive models (ANOVA), regression on the mean (Yates and Cochran 1938; Finlay and Wilkinson 1963), additive main effects and multiplicative interaction (AMMI) models (Gauch Jr. 1992) and the genotype + genotype × environment (GGE) model (Yan et al. 2000). Since they only require a two-way table of means as input, these models are considered to be good for descriptive and explorative purposes, but not for explaining G × E (Malosetti et al. 2013). The second group of models includes factorial regression, partial least squares regression, structural equation models and mixed effect models. Factorial regression allows the use of environmental or genotypic variables as covariates to explain G × E but has the limitation that only permits one dependent variable at a time (Vargas et al. 2007). Another limitation of factorial regression is its difficulty in dealing with multi-collinearity when several covariates are used (Vargas et al. 1999). For these cases, partial least squares regression is a more convenient approach, as it can easily handle multiple explanatory variables (Vargas et al. 1999). When the cause-effect analysis of G × E is aimed for, partial least squares regression becomes inadequate, and methods such as structural equation modelling are more suitable (Vargas et al. 2007).

Mixed-effect models are one of the most used approaches for analysing G × E, and they are usually implemented using either single-stage or two-stage analysis (Möhring and Piepho 2009). Single-stage models analyse data from individual plots, in which the residual effects and the G × E effects are estimated simultaneously (Smith et al. 2005). In contrast, two-stage models include a first stage in which design features and spatial variation are modelled using data from individual trials. Next, the second stage involves fitting an overall mixed model to the genotype by environment adjusted means obtained from stage 1 (Malosetti et al. 2013; Smith et al. 2005). The analysis can be extended to more than two stages, in which case the approach is more commonly known as stage-wise analysis (Piepho et al. 2012a; Damesa et al. 2017). Although single-stage analysis is preferred from a theoretical point of view, two-stage analysis is less demanding in terms of computation requirements and provides similar results to single-stage when appropriate weights are selected (Malosetti et al. 2013). Therefore, most of G × E models are implemented using a two-stage approach (Malosetti et al. 2013).

There are situations where non-parametric methods, such as rank-based methods, are more convenient (Elias et al. 2016). This type of non-parametric methods has been used mainly to rank genotypes at specific locations (Elias et al. 2016) and has a set of advantages that include no specific modelling assumption about the distribution of the effects and are easy to implement and interpret (Huehn 1990). Non-parametric models are considered a useful option when the interest is focused on the ranking of genotypes rather than to evaluate the level of difference on performance between genotypes (Brancourt-Hulmel et al. 1997). In the context of selection in breeding and evaluation programs, Huehn (1990) considered the rank order of genotypes to be the most important information.

Other G × E models go beyond statistical analysis and integrate knowledge on crop physiology and expert assessments. For instance, Theobald et al. (2002) proposed the use of a Bayesian model to incorporate expert knowledge about the analysed crop. In a bibliometric analysis, van Eeuwijk et al. (2016) identified an important growth in the application of both mixed models and crop growth models, especially after 2005. A crop growth model incorporates plant physiological aspects, along with the genotype and environment, in the analysis of interactions that produce a phenotype (van Eeuwijk et al. 2016). Furthermore, crop growth models also allow to consider the effect of cropping systems (intercropping, fertility management, etc.) on G × E (Jeuffroy et al. 2014).

One of the goals of crop growth models for variety evaluation in multi-environment trials is to improve the characterization of the environment (Jeuffroy et al. 2014). For example, Tesfaye et al. (2016) combined geospatial analysis with crop modelling (1) to characterize a maize growing environment in Southern Africa (Malawi, Mozambique, Zambia and Zimbabwe) and (2) to evaluate the variety performance of five new drought-tolerant varieties across the aforementioned region. The environmental characterization was conducted using a standardized precipitation index, and it focused on the frequency of drought occurrences rather than drought severity (Tesfaye et al. 2016). To evaluate the variety performance of new varieties, maize yields were simulated using the Crop Estimation through Resource and Environment Synthesis (CERES)-Maize model. Simulated relative yields of five drought-tolerant varieties outperformed the commercial check variety across many environments, but not in all (Tesfaye et al. 2016).

Jeuffroy et al. (2014) reviewed the use of crop growth models in variety performance prediction and concluded that although their use is increasing, they are still not mainstream for variety evaluation. For mechanistic models to have predictive power to distinguish between varieties, information is needed on the processes or the underlying genotypic factors that give rise to these differences. Some information can be derived from existing trial data through model fitting, but overfitting often occurs. Acquiring additional data to estimate crop model parameters directly is often costly or not possible retrospectively in a data synthesis context. Jeuffroy et al. (2014) argue that cost-benefit considerations to assess the value of additional information are important. On the one hand, crop growth modelling generally focuses on a narrow set of variables (mostly yield). Yield is an important input into crop variety recommendations, but other aspects cannot be ignored, including the user perspective (see Section 4.2). Their relative complexity makes their application often difficult. One possibility is to use (at least initially) very simple crop models and build up their complexity gradually (Shorter et al. 1991). Another option is to generate intermediate variables that can be used in statistical models or machine learning approaches (see Feng et al. 2019).

### Evaluation in target environments and including user requirements

The current challenges in agricultural production are more likely to be addressed by locally adapted solutions that consider both environmental and socioeconomic information (Van Etten et al. 2016). The socioeconomic context of the target environment should be considered to match both farmer and consumer preferences. Socioeconomic data like human population, welfare and transportation infrastructure have been proposed for targeting genotypes to environments, but such recommendations mostly concerned logistics planning on germplasm deployment (Hyman et al. 2013). While this kind of socioeconomic data is indeed important, other types of data, such as consumer preferences, should also be considered. For example, Desclaux et al. (2008) proposed that, in addition to the usual biophysical and management factors, the environment should be a wider concept that also includes actors, markets, regulations and societal dynamics.

Participatory on-farm trials aim to take the variety trials closer to the target environments and user requirements (Ceccarelli and Grando 2007). On-farm trials can provide much information, ranging from biophysical performance to economic assessment (Franzel and Coe 2002). In this type of trials, the concept of environment in a G × E analysis is extended to include socioeconomic factors, besides the usual biophysical variables (Coe 2002). Data collected from on-farm participatory trials is often in the form of ratings or rankings, requiring different statistical models to conduct G × E analysis (Coe 2002). For example, Coe (2002) proposes to analyse ratings using proportional odds, and rankings with the Bradley-Terry model (Bradley and Terry 1952). An extended Bradley-Terry model can incorporate covariates (Coe 2002; Dittrich et al. 1998). As discussed before, the possibility of including covariates is especially relevant when location-specific information is to be extracted from the experimental data. It has been shown that environmental covariates can improve predictions of variety performance (Piepho 2000; Piepho et al. 1998). For data synthesis, in cases when environmental data is not collected as part of the crop trial, environmental covariates can be linked to experimental data through geolocation, as shown by Lobell et al. (2011), van Etten et al. (2019) and others.

An extended version of the Plackett-Luce model (Plackett 1975; Luce 1959) recently implemented by Turner et al. (2020) includes the use of model-based recursive partitioning (Strobl et al. 2011), allowing the incorporation of covariates for predicting rank orders. Hence, subgroups of rankings with significantly different worth parameters are identified based on covariates (Turner et al. 2020). van Etten et al. (2019) recently used this model to analyse data from on-farm participatory trials in three countries, successfully identifying environmental covariates that consistently influence variety performance across several seasons.

### Multi-dimensional assessment for decision-making

An overall evaluation of varieties requires joint analysis of several traits, from both biophysical and socioeconomic perspectives. Different approaches have been proposed to handle multi-criteria prioritization on ranking varieties according to different traits of interest. Abeyasekera et al. (2002) developed a methodology to combine scores and rankings assigned by farmers evaluating bean (*Phaseolus vulgaris*) varieties, using a weighted index, which allows the farmer’s preference to be captured, thus combining multiple criteria. The work of Abeyasekera et al. (2002) considered the farmers’ preferences in terms of not only agronomic criteria (yield, pest resistance, etc.) but also non-agronomic criteria, such as taste, marketability and cooking time. Waldman et al. (2014) used choice experiment models to estimate farmers’ preferences of perennial pigeon pea. Smith and Fennessy (2011) applied the PAPRIKA (Potentially All Pairwise RanKings of all possible Alternatives) method (Hansen and Ombler 2008) to assess the relative importance of traits on the improvement of perennial pasture species. The PAPRIKA method asks participants to compare pairs of options (varieties) and select one. It assumes full transitivity to reduce the number of pairs compared. In other words, when A > B and B > C, the model assumes A > C. An alternative method for priority setting is AgroDuos (Steinke and van Etten 2017), which is similar to PAPRIKA but integrates the concept of gamification to increase participants’ engagement (Deterding et al. 2011), while it does not require interactive updating of questions and can therefore be used without a digital device or Internet connection.

Farmers’ comprehensive evaluations of the total value of a variety can also be derived from on-farm trials. For example, the ‘tricot’ (*triadic comparisons of technologies*) approach proposed by Van Etten et al. (2016) integrates farmers’ feedback on variety evaluation as a ranking of varieties, based on their overall appreciation of the varieties. In the tricot approach, each farmer receives three packages of seeds, each with a different variety of the crop (Van Etten et al. 2016). Each farmer ranks the three varieties from best to worst, according to overall performance, considering traits such as pest resistance and yield (Van Etten et al. 2016). Rankings of varieties directly evaluated by farmers in on-farm trials are aggregated by rank aggregation models (see Section 5.1).

**Fig. 4:**
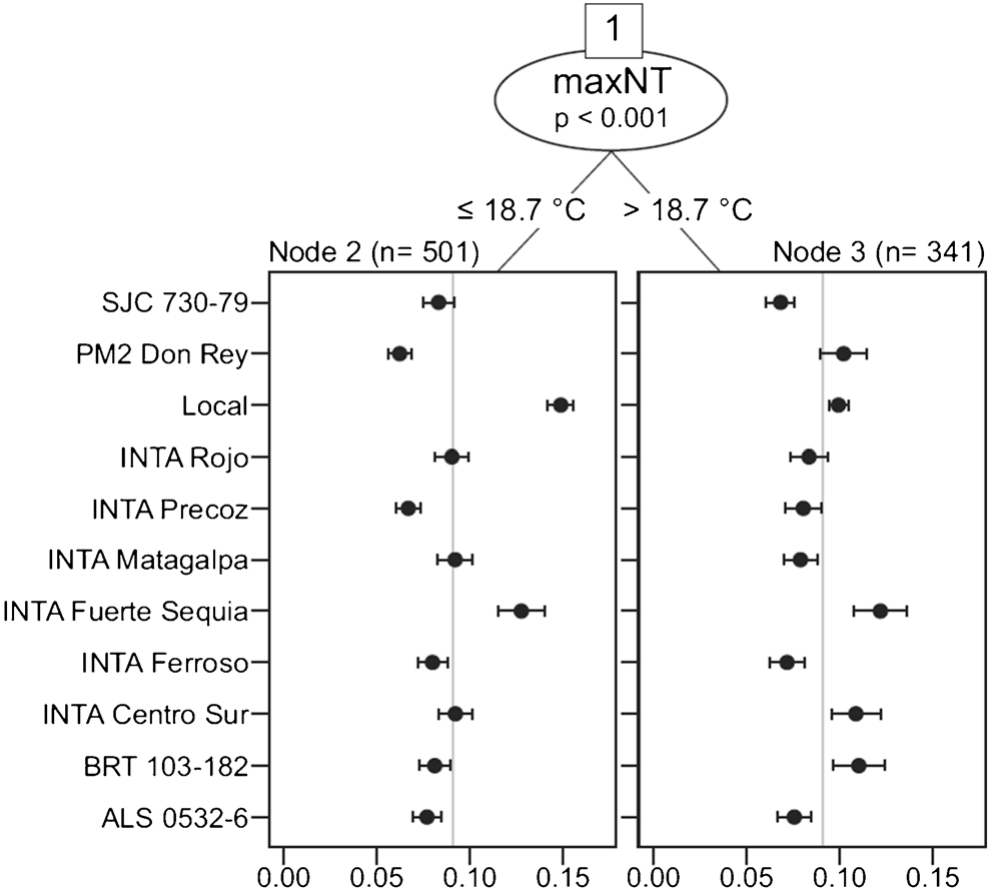
Plackett-Luce Tree of farmer-participatory tricot trial data in Nicaragua. The probability of each variety to perform better than the others in the trial is presented on the horizontal axis. Grey vertical line represents the average probability of better performance (1/number of evaluated varieties). From the study of van Etten et al. (2019, p. 4196, CC BY-NC-ND)

Recent work of van Etten et al. (2019) is an example of how a rank-based model was applied to consider several criteria, such as disease resistance, yield and farmer preferences into a single judgement, in combination with local environmental conditions in the analysis crop variety trials. The work of van Etten et al. (2019) includes three independent studies in three countries: Ethiopia, India and Nicaragua. For brevity, we focus on the case of Nicaragua, where varieties of common bean were evaluated in 842 plots. An extended version of the Plackett-Luce model, implemented in the PlackettLuce package (Turner et al. 2020), was fitted for the trial data collected from on-farm trials, which were established following the tricot approach (van Etten et al. 2019). The Plackett-Luce model estimates a worth parameter that represents the log probability of each evaluated element (a crop variety in this case) to be ranked first. Environmental conditions of the trial locations were included into the model using climatic indices (Table 1) as model covariates, through model-based recursive partitioning, implemented in the PlackettLuce package as Plackett-Luce Trees. The use of climatic variables as model covariates led to the identification of environmental factors that influenced the probability of a variety performing better than the other varieties tested in the trials (see Fig. 4 for an example).

The evaluations mentioned above focus on average performance of varieties, but other approaches focus on the variation in performance across seasons to assess farmers’ risks. It has been shown that multi-environmental trial data from several seasons can be used to propose variety portfolios to reduce risk and maximize farmers’ profits (Nalley et al. 2009; Nalley and Barkley 2010; Sukcharoen and Leatham 2016). These studies all focus on yield as the main evaluation criterion. Fadda and van Etten et al. (2019) proposed the adaptation of portfolio selection theory from financial asset management field, based on the portfolio management method developed by Dembo and King (1992). Instead of recommending a single variety, a portfolio of varieties is recommended based on calculations of the expected regret (Fadda and van Etten 2019). This method does not require absolute (yield) data and can also be applied to ranking data. This is interesting for progress in data synthesis, as ranking methods can play a role in combining datasets from different sources (see Section 5.1).

## Data synthesis approaches

In the previous section, we reviewed methods for the analysis of different data types used for crop variety evaluation. In addition to that, data synthesis involves integration of datasets from heterogenous sources. For instance, datasets come from several research programs, each one with diverse types of data formats, measurement units and experimental designs.

Data synthesis for crop variety evaluation has followed two main lines of research: rank aggregation and network meta-analysis. In the remaining part of this section, we review relevant examples from both rank aggregation and network meta-analysis, to finally weigh their advantages, disadvantages and existing gaps towards a data synthesis methodology for crop variety evaluation.

### Rank aggregation methods

Rank aggregation methods are rank-based non-parametric statistical methods that allow for aggregation of results from individual studies to obtain one consensus ranking (Lin 2010; Yu et al. 2019). They have been applied to several fields including advertisement research, psychology, Internet search engines and biological studies (Lin 2010). Rank aggregation methods are suitable for high-level meta-analysis, where aggregation of different raw data is not feasible (Lin and Ding 2009). They also provide more statistical power than individual analyses (Simko and Pechenick 2010; Lin 2010). This coincides with one of the widely argued characteristics of meta-analysis (Cohn and Becker 2003).

Simko and Pechenick (2010) proposed to use rank aggregation methods to combine heterogenous data from independent plant breeding trials. Simko and Linacre (2010) demonstrated how the Rasch model (Rasch 1960) can be used to combine heterogeneous data. The Rasch model is, in principle, very similar to the Luce model (Rasch 1960; Luce 1959).

Simko and Linacre (2010) presented four different real datasets as case examples; for brevity, we only focus on one of the datasets, containing data of potato chip quality evaluations. The analysis of this dataset implies two main constraints already mentioned in Section 3: (1) data measurements in different rating scales and (2) only partial overlap among tested varieties. Potato chip quality data were collected from online databases of 10 different laboratories. As we described in Section 2, assessment of food quality and consumer preferences can be done using several rating scales. In the case of quality assessments of potato chips, Simko and Linacre (2010) explained that it is a common practice that each laboratory uses a different rating scale such as one of the following: (1) a rating scale of 5, 9 or 10 categories (the number is subjectively selected by each laboratory; lower values indicate higher quality of potato chip); (2) a measurement of the potato chip colour using specialized equipment with values ranging from 0 to 100 (higher readings indicate a lighter colour of potato chips, which is a desired trait); and (3) a percentage of chips passing a given quality test defined by the laboratory. In this example, it was not specified which rating scale was used by each laboratory in each test, but indeed different ranges of values exist across the different tests.

The data of potato chip quality assessments collected from 10 different laboratories were aggregated into one dataset. The aggregated dataset contained 63 cultivars over 157 trials, with only partial overlap among evaluated cultivars. For instance, only one cultivar was evaluated in 154 trials, while only seven cultivars were evaluated in a single trial (Simko and Linacre 2010). The resulting matrix contains 994 data points, around 10% of the expected total data points (9891) that would have resulted if all the varieties had been evaluated in all the trials (Simko and Linacre 2010). The original ordinal ratings are replaced for relative rankings (Simko and Linacre 2010). The relative rankings were used to calculate an overall performance rating, by means of an extended version of the Rasch model (Simko et al. 2012; Linacre and Wilson 1992). In this case, the extended version of the Rasch model allowed to compare 63 cultivars, even when not all were tested in the same trial.

Interestingly, rank aggregation has also found a direct application in variety trials, such as the work of van Etten et al. (2019) presented in Section 4. The successful application of rank-based methods in both trial analysis and meta-analysis shows that this is an interesting way forward in data synthesis for variety evaluation.

### Network meta-analysis

Commonly used meta-analysis methodologies, especially in the medical sciences, are often based on pairwise comparison of treatments, usually in the form of an intervention against a control or placebo (Lumley 2002; Tonin et al. 2017). Network meta-analysis (Lumley 2002) allows the comparison of multiple treatments, even when some of them have never been compared directly in trials (Tonin et al. 2017). Although network meta-analysis is commonly used in medical sciences, it has also been used recently in other fields such as plant pathology (Madden et al. 2016). Network meta-analysis is also known by several other terms, such as ‘multiple treatments meta-analysis’ and ‘mixed treatment comparison’, which are often used interchangeably (Salanti 2012).

**Fig. 5:**
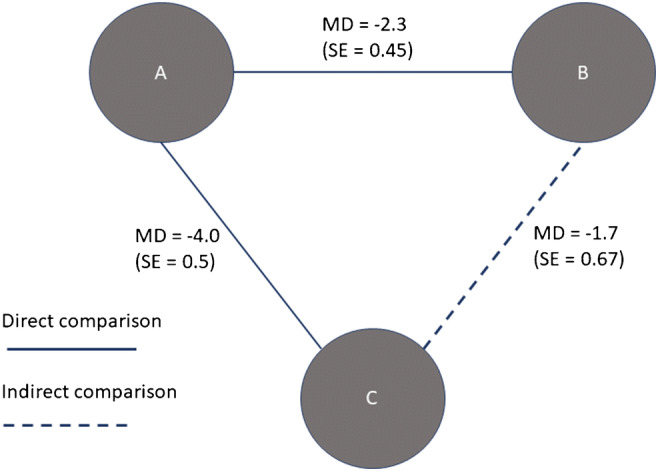
Example of a network of treatments (varieties) allowing direct and indirect comparisons. Adapted from Dias and Caldwell (2019). MD, mean difference

As explained by Tonin et al. (2017), the approach evolved from the initial work of Bucher et al. (1997) on ‘adjusted indirect treatment comparison’, which was called ‘network meta-analysis’ after the improvements made by Lumley (2002), and later evolved to ‘mixed treatment comparison’ by Lu and Ades (2004). A distinctive characteristic of network meta-analysis is the case when both direct and indirect comparisons are available for a given pair of treatments. In this case, evidence from both direct and indirect comparisons is used to do a mixed treatment comparison (Fig. 5), hence the alternative name (Dias and Caldwell 2019). For more details about related terminology on mixed treatment comparisons, we refer to Salanti (2012) and Coleman et al. (2012).

Network meta-analysis can be implemented with two different types of models: (1) contrast-based models, also known as conditional models, in which the treatment effects per trial are estimated as a contrast relative to a baseline treatment to subsequently analyse all the contrasts across studies, and (2) arm-based models, also known as unconditional models, in which the treatment summaries per trial are analysed in a two-way linear mixed model (Piepho et al. 2012b; Madden et al. 2016). Arm-based models are commonly applied for the analysis of multi-environment crop variety trials (Section 4) (Piepho 1997; Albert and Makowski 2018; Damesa et al. 2017). As explained in Section 4, it is possible to use single-stage or two-stage analysis in linear mixed models, although for network meta-analysis, a single-stage analysis might be constrained by the availability of data from individual primary studies, rather than usual summary results such as the estimated effect sizes (Madden et al. 2016).

Both frequentist and Bayesian approaches can be applied to network meta-analysis (Tonin et al. 2017), although the Bayesian approach seems to be more popular (Piepho et al. 2012b). Network meta-analysis usually includes the use of network diagrams, where the nodes represent the compared elements (e.g. treatments or varieties), and the lines (edges) connecting the nodes represent the direct comparison of elements, to evaluate network connectivity. This is relevant in network meta-analysis, especially because poorly connected networks might provide less reliable results compared to a strongly connected network (Tonin et al. 2017). It is also possible the computation of ranking probabilities for each treatment to be assigned a particular position in a ranking from best to worst (Tonin et al. 2017).

Based on yield data obtained from 28 published papers selected through a systematic literature review, Laurent et al. (2015) applied both direct and indirect comparisons in a meta-analysis for ranking crop species based on yield. Direct comparisons compare crops which were grown at the same site and in the same year, whereas indirect comparisons compare crops grown at different sites or in different years, using a third crop grown at all sites as a reference (Laurent et al. 2015). In this case, only results from experimental sites were considered (no farmers’ fields), resulting in a database containing 856 records of yield for 36 crop species (Laurent et al. 2015). Mean yield was estimated using a linear mixed effect model, with a log transformation to normalize the yield data (Laurent et al. 2015). For the direct comparison, four crops species (*Miscanthus* × *giganteus*, *Panicum virgatum*, *Triticosecale*, *Salix*) were selected to be used as reference crops, as they were included in the higher number of comparisons with other crops for the same site-years (Laurent et al. 2015). A model was fitted for each reference crop using restricted maximum likelihood. Then, yield ratios of the mean yield of each evaluated crop (except reference crops) to the mean yield of a reference crop grown in the same site and year were calculated (Laurent et al. 2015).

Since direct comparison allows to compare only a limited number of species, indirect comparison was used to compare the yields of a crop of interest, *Miscanthus* × *giganteus*, to yields of crops that were not grown in the same site-years as the crop of interest. Three reference crops were selected for the indirect comparison: *Panicum virgatum*, *Triticosecale* and *Salix*. Therefore, *Miscanthus* × *giganteus* was compared to crops not grown in the same site-years, by indirect comparison using the reference crops, allowing to include more crop species than using direct comparison only.

Albert and Makowski (2018) recently published a paper describing the use of Bayesian mixed treatment comparison models for ranking crop species. According to Albert and Makowski (2018), the dataset used is the same as that analysed in Laurent et al. (2015), although they also indicate that 639 yield observations were analysed, which are less than the 856 yield data observations analysed in Laurent et al. (2015). Mixed treatment comparison combines direct and indirect evidence (Dias and Caldwell 2019). Five different models were fitted (Table 4), of which four were contrast-based models and one was an arm-based model. According to Albert and Makowski (2018), the Bayesian contrast-based models (1 to 4) are variants of the model presented by Dias et al. (2010), while the arm-based is a Bayesian two-way model. The model estimation was done using Markov chain Monte Carlo simulations, while model assessment was made using the deviance information criterion (DIC), in which the models with the lowest DIC are preferred (Albert and Makowski 2018). Compared to the rankings obtained by Laurent et al. (2015) using direct and indirect comparison, the results are very similar for the two species with higher yields (*Pennisetum purpureum and Arundo donax*) when compared against *Miscanthus* × *giganteus*.

**Table 4:** Description of models used by Albert and Makowski (2018)

Model number	Model type	Effect	Variance	DIC
1	Contrast-based	Fixed	Common residual	912
2	Contrast-based	Random	Common residual	348
3	Contrast-based	Random	Species-specific residual	287
4	Contrast-based	Random	Study-specific residual	214
5	Arm-based	Two-way model		348

### Assessment of available data synthesis methods

The methods reviewed above address the challenge of combining crop variety trial data from multiple and independent sources. In Section 3, we presented a set of challenges identified by Simko and Pechenick (2010) that arise when aiming to combine data from different trials. Here, we assess both rank aggregation and network meta-analysis as solutions to those problems. Additionally, we provide an overview of the relative strengths and weaknesses of data synthesis methods.

#### Partial overlap in evaluated accessions between trials

The problem of partial overlap in the varieties evaluated across trials can be solved by exploiting the capacity of rank aggregation methods to handle partially ranked lists, although the specific approach depends on the particular rank aggregation method. For example, some models are based on pairwise comparison such as the Bradley-Terry model, while others allow multiple comparisons, such as models based on the Plackett-Luce model. In the case of network meta-analysis, the problem of partial overlap is solved by indirect comparison. For example, in Fig. 5, items B and C are indirectly compared with A. Examples are Laurent et al. (2015) and Albert and Makowski (2018) who used reference crop species to allow the comparison of crop species not tested in the same trial.

#### Measurements based on different rating scales or different methods

Rank aggregation methods solve the problems of measurements in different rating scales or traits evaluated with different methodologies, replacing the original raw data from each trial by relative rankings (Simko and Piepho 2011; Simko et al. 2012). In the work of Laurent et al. (2015) and Albert and Makowski (2018) this was no issue, however, because the data from the different yield studies were in the same units, tons of dry matter per ha per year (Laurent et al. 2015). Network meta-analysis and meta-analysis, in general, can deal with measurements in different units by estimating either the standardized mean difference or the response ratio (Borenstein et al. 2009; Makowski et al. 2019; Murad et al. 2019).

#### Relative strengths and weaknesses of data synthesis methods

There are a few studies applying either rank aggregation or network meta-analysis to crop variety evaluation. Future studies will need to consider the relative merits of each.

Network meta-analysis can provide absolute values (yield differences in tons per hectare), which is difficult to obtain with rank-based models. Even so, the item ‘worth’ estimated by the rank-based methods is linearly correlated with the underlying latent variable (for example, yield) (Coe 2002; Fadda and van Etten 2019). Also, in theory, it should be possible to combine ranking data and continuous variables in the same model (Böckenholt 2004), but this is still challenging in practice, as such models have not been implemented in general use software.

A useful output that can be obtained from both rank aggregation and network meta-analysis is ranking probabilities, the probability of each variety to be ranked first. Ranking probabilities are related to the concept of *reliability* in plant breeding, the probability of outperforming a check variety (a reference; for example a previously released variety, commonly used variety or market leader). The concept of reliability was proposed by Eskridge (1990) in the context of crop improvement as a ‘safety-first’ approach, with subsequent applications by Eskridge and Mumm (1992) and Eskridge (1997).

Data synthesis approaches should consider the ease of use and interpretation by decision-makers in crop variety evaluation. In that sense, the complexity of network meta-analysis can lead to confusion on model implementation and interpretation (Madden et al. 2016). This complexity might be a barrier to its wider adoption as a tool for data synthesis in crop variety evaluation, just like the low level of expertise of users, is limiting the uptake of more sophisticated G × E analysis methods (Lecomte et al. 2010). Rank aggregation methods might be easier to implement but have implicit trade-offs such as information loss and less power to detect existing differences if compared to parametric methods (Simko and Linacre 2010; Whitley and Ball 2002; Sabaghnia 2016).

## Conclusions and recommendations

We structure this section around three main statements based on our review, which derive conclusions from our main findings and translate these into recommendations.

### Elements for a data synthesis approach are available and aligned around ranks and reliability

Based on our review, we assert that the main elements are available for data synthesis as an overarching approach that integrates different components, such as data, models and knowledge from experts (farmers and breeders), to efficiently extract useful information to support decision-making. We remarked in Section 5 data synthesis methods that have been tested, exist and can integrate well with existing trial analysis approaches. In particular, rank-based approaches fit within a conceptual framework to analyse variety superiority based on reliability (probability of outperforming a check). A rank-based framework would be able to make versatile use of data from different sources, without complex transformations or doubtful assumptions, and would facilitate the integration of objective measurements and preference data.

### Data synthesis should progress from general to specific and from simple to complex

Explicit crop growth modelling has been proposed more than once as a way forward to integrate different types of data into a single conceptual framework for the evaluation of variety performance. However, model building starting from a detailed crop model is not parsimonious and does not build up complexity in a gradual way. For many crops, growth models are not available, hence requiring a large upfront investment in basic (eco)physiological research to enable model building. Also, as shown in Section 4, it seems that progress in this field is mainly theoretical, and that practical advances are limited. Even for the attempts that result in generalizable results, the focus is solely on yield and excludes user perspectives. For crop variety evaluation, it seems more logical to start with the ‘big picture’ and work down to the details based on better information indicating where the largest gain in accuracy can be obtained (Section 4). This may involve some type of explicit, physiological modelling, but perhaps of a limited number of aspects, not requiring a fully fledged crop growth simulation model. Therefore, we think that a further investment in simpler methods is warranted. This may be less stimulating from a basic research point of view but may give rise to new questions and priorities and give a better sense of the societal relevance and external validity of data synthesis efforts.

### Use cases can spur further data sharing and model development

Our review shows that engaging the research community in data sharing is a major challenge (Section 3). Most efforts, however, have focused on the supply side: by encouraging/obligating researchers to share data and by providing the infrastructure to do so. While these efforts are certainly important, in crop science, few concomitant efforts have looked at the prospects of successful use of shared data that would drive citations of data papers, shape collaborations around data analysis, and increase researchers’ motivation for further sharing. Success may at least partially be the result of a siphon effect: some early use cases can perhaps inspire other researchers to engage in sharing and start a virtuous cycle, as described in Section 3. Therefore, investment in a few use cases that use relatively simple methods to show the potential benefits of data synthesis for crop variety evaluation is needed. Our review has shown that those methods are available in principle (Section 5). Even so, they need a modest investment to be adapted and demonstrated for this field of application. Next steps would involve stepwise refinements to address components of variety performance that substantially improve the accuracy of predictions. Close collaboration with the decision-makers interested in such evaluations could also spur further interest in this area of research and demonstrate the relevance of further investment.
